# Disrupted Neural Regeneration in Dry Eye Secondary to Ankylosing Spondylitis—With a Theoretical Link between Piezo2 Channelopathy and Gateway Reflex, WDR Neurons, and Flare-Ups

**DOI:** 10.3390/ijms242015455

**Published:** 2023-10-22

**Authors:** Balázs Sonkodi, László Marsovszky, Anita Csorba, Attila Balog, Bence Kopper, Anikó Keller-Pintér, Zoltán Zsolt Nagy, Miklós D. Resch

**Affiliations:** 1 Department of Health Sciences and Sport Medicine, Hungarian University of Sports Science, 1123 Budapest, Hungary; 2 Department of Ophthalmology, Semmelweis University, 1085 Budapest, Hungary; marsovsz@gmail.com (L.M.);; 3 Department of Rheumatology and Immunology, Albert Szent-Györgyi Medical School, University of Szeged, 6725 Szeged, Hungary; 4 Faculty of Kinesiology, Hungarian University of Sports Science, 1123 Budapest, Hungary; 5 Department of Biochemistry, Albert Szent-Györgyi Medical School, University of Szeged, 6725 Szeged, Hungary

**Keywords:** dry eye disease, ankylosing spondylitis, autoimmune disease, Piezo2 channelopathy, Piezo1, Th17/Treg imbalance, gateway reflex, WDR neurons

## Abstract

This study aimed at analyzing the corneal neural regeneration in ankylosing spondylitis patients using in vivo corneal confocal microscopy in correlation with Langerhans cell density, morphology, and dry eye parameters. Approximately 24 ankylosing spondylitis subjects and 35 age- and gender-matched control subjects were enrolled. Data analysis showed that all corneal nerve-fiber descriptives were lower in the ankylosing spondylitis group, implicating disrupted neural regeneration. Peripheral Langerhans cell density showed a negative correlation with nerve fiber descriptions. A negative correlation between tear film break-up time and corneal nerve fiber total branch density was detected. The potential role of somatosensory terminal Piezo2 channelopathy in the pathogenesis of dry eye disease and ankylosing spondylitis is highlighted in our study, exposing the neuroimmunological link between these diseases. We hypothesized earlier that spinal neuroimmune-induced sensitization due to this somatosensory terminal primary damage could lead to Langerhans cell activation in the cornea, in association with downregulated Piezo1 channels on these cells. This activation could lead to a Th17/Treg imbalance in dry eye secondary to ankylosing spondylitis. Hence, the corneal Piezo2 channelopathy-induced impaired Piezo2-Piezo1 crosstalk could explain the disrupted neural regeneration. Moreover, the translation of our findings highlights the link between Piezo2 channelopathy-induced gateway to pathophysiology and the gateway reflex, not to mention the potential role of spinal wide dynamic range neurons in the evolution of neuropathic pain and the flare-ups in ankylosing spondylitis and dry eye disease.

## 1. Introduction

Ankylosing Spondylitis (AS) is a chronic, progressive inflammatory disease that primarily affects the axial joints and eventually transforms the vertebra into an enigmatic clinical picture of kyphotic posture, accompanied by inflammation of the sacroiliac joints and spine, especially in the vicinity of the lumbar 5 (L5) spinal segment. Kyphosis and other postural changes may further affect balance and proprioception in AS. Inflammatory back pain, stiffness, and swelling are due to enthesis, osteitis, synovitis, and pathological new bone formation in AS [1]. The etiology of AS has been unknown for more than 100 years, like in the cases of delayed onset muscle soreness (DOMS) [2] and amyotrophic lateral sclerosis (ALS) [3], but genetic and environmental risk factors are implicated in the pathophysiology, leading to the dysregulated activation of the innate and adaptive immune systems and resultant immune cell imbalance in the inflammatory processes of AS [4].

Dry eye disease (DED) is a multifactorial disease of the ocular surface characterized by a loss of homeostasis of the tear film and accompanied by ocular symptoms, in which tear film instability and hyperosmolarity, ocular surface inflammation and damage, and neurosensory abnormalities play etiological roles [5,6,7]. Interestingly, DED has been primarily implicated in conjunction with autoimmune diseases for more than three decades [6]. The co-existence of autoimmune conditions and ocular complications, including dry eye disease, has been well documented in the literature [8], where AS is significantly correlated with the incidence of DED [9]. Uveitis is the most common intraocular inflammatory eye condition that may be brought on during any stage of AS, but we know very little about corneal involvement in the cascade of events leading to distressed ocular surfaces and dry eyes. One of the key dysregulated processes that may take place in the initiation of dry eye disease could be related to the ion channels operational on the ocular surface [10].

Indeed, the principal mechanotransductory ion channel for proprioception is shown to be the evolutionarily highly conserved Piezo2 transmembrane protein [11]. This ion channel also participates in indentation/compression, stretching, shear stress, and vibration detection. Furthermore, it has been postulated that the microdamage of the Piezo2 ion channel in an autologous way at proprioceptive neuron terminals could be one principal gateway between physiology and pathophysiology, or, in other terms, the long-suspected primary damage [12]. In addition, it is suggested that Piezo2 channelopathy is also a principal transcription activator [12]. This initial neural Piezo2 microdamage is suspected in numerous autoimmune diseases that could evolve into an autoinflammatory chronic disease condition as a consequence of the chronification of the proposed transient Piezo2 channelopathy in association with certain genetic and environmental risk factors [13,14].

AS is also called axial spondyloarthritis (axSpA) or Bekhterev’s disease. It is interesting to note that Vladimir Bekhterev, a Russian physician who is often cited as the first physician to describe AS more than 100 years ago, practiced medicine in the field of neurology [15]. It is noteworthy that the proposed proprioceptive terminal Piezo2 channelopathy is likely to be associated with the observed impairment of the static phase firing encoding of the stretch reflex [12,16,17,18] and this impairment probably affects not only proprioception but postural control as well [3,17,19,20]. Furthermore, recent neurocentric articles are emphasizing the relevance of stress-related axial non-contact, compressive, stretch-related, or vibratory mechano-energetic neural microdamage in certain musculoskeletal [13,17,19] pathophysiologies. It is noteworthy that mechanical stress, microdamage, and lost mechanotransductory signaling are also implicated at these sites in AS [21], especially in the vicinity of the L5 segment. Accordingly, the current authors postulate that the primary damage in AS could also be associated with Piezo2 channelopathy due to stress-related excessive mechanotransduction in encapsulated mechanoreceptors of neural endings in the spinal facet joints, in a similar fashion as is suspected in the initial neural microdamage of osteoporosis [13]. Correspondingly, the co-occurrence of osteoporosis with AS is common [22]. However, the critical pathomechanistic molecular roadmap downstream will be different due to different disease-specific genetic and environmental risk factors. In support of this theory, the cervical spine is one heavily affected region in AS, and it has recently been demonstrated that cervical proprioception is indeed impaired in AS [23], like in RA [24]. It is no surprise since Piezo2 channelopathy is suggested to attract more neuro-energetic resources in the affected spinal segment as a compensatory and protective mechanism, e.g., in the form of exaggerated contractions, but as a consequence, the highest spinal neuro-energetic proprioceptive capacity, namely the cervical region, will be negatively affected due to resource limitations of the overall proprioceptive system [3,16,20] or even more precisely the Piezo system [10].

Piezo2 ion channels are known to be inactivated when they go through hyperexcitation, and this inactivation is within homeostasis [25]. However, the Piezo2 channelopathy theory postulates that this gating machinery of Piezo2 could be microdamaged due to further prolonged excessive mechanotransduction under allostatic stress, leading to an influx of leakage subthreshold Ca^2+^ currents [16,17]. It is noteworthy that the suggested excessive mechanotransduction of Piezo2 containing proprioceptive sensory afferents, also found in muscle spindles and tendons, in the presence of a gain-of-function Piezo2 mutation (which is certainly an inherited form of Piezo2 channelopathy) indeed causes joint stiffness and contractures and disrupts musculoskeletal developments [26], as was theorized earlier in mussels [3,17]. The authors of this manuscript suggest that Piezo2 channelopathy and the resultant unwanted subthreshold Ca^2+^ currents could propel the pathomechanism towards enthesis, not to mention contributing to pain sensation and even to neuropathic pain. It is important to note that the loss-of-function mutations in Piezo2 cause loss of pain and sensitization [27] and Piezo2 also has a role in the evolution of mechanical allodynia leading to neuropathic pain [28] by participating in the response of Aδ fibers to noxious mechanical stimuli in bone nociception [29]. Even more critically, Piezo channels indeed have a role in neural regeneration, axonal growth, and neurite outgrowth as a consequence of injury [30] and physical stimulation involving smart piezoelectric nano-biomaterials is gaining ground as potential procedures for neural regeneration [31,32].

A further indication of neural involvement in AS aside from pain is that AS patients have more fatty degeneration, atrophy, and denervation in their paraspinal muscles [33], in association with subclinical neurological complications [34]. Correspondingly, our team has recently proved that regeneration of corneal nerves is also disrupted in DED secondary to autoimmune diseases [35,36]. The disruption in corneal neural regeneration is theorized to be initiated by chronic Piezo2 channelopathy-induced impaired Piezo2-Piezo1 crosstalk, hence the constantly activated transcription process and disrupted, but “kept alive”, wound healing [10,35,36]. Moreover, the higher prevalence of DED in certain autoimmune conditions could be owed to further indirect causes, like proteoglycan depletion and other ion channel links, like K_2_p and Kv1.3, in addition to the underlying autologous chronic Piezo2 channelopathy [35,36]. It is important to note that the bidirectionality of the pathophysiology that could lead to Piezo2 channelopathy in DED is emphasized, meaning that prolonged overstimulation of Piezo1 mechanotransduction could also lead to Piezo2 channelopathy, like in the case of direct non-contact neural microdamage of Piezo2 [10].

Even more importantly, the natural killer T cells (NKT) cells are shown to be elevated in the transient form of this proposed proprioceptive terminal Piezo2 channelopathy through the Hsp70/TLR4/IL-6/TNF-α pathway [37]. The chronification of this process will lead to the reduction of these cells in autoinflammatory conditions and autoimmune diseases [13,14], like in AS [38]. It was suggested earlier that the chronic Piezo2 channelopathy-induced impaired Piezo2-Piezo1 crosstalk will deplete certain proteoglycans [36]. Interestingly, one proteoglycan, namely syndecan-1, negatively regulates the homeostasis of Interleukin-17 (IL-17) producing NKT cells (NKT17) [39], hence the chronic depletion of syndecan-1 might lead to lost regulation of NKT cells and the resultant depletion of them in AS. These NKT17 cells and γδ T cells (Tγδ17) were identified with sdc1 markers [39]. Syndecan-1 also binds other cytokines, e.g., IL-8 [40] or IL-34 [41], via its glycosaminoglycan chains and functions as a co-receptor modulating cytokine signaling. Therefore, the increased IL-17 production in syndecan-1 knockout mice [39] might be a compensatory mechanism after decreased IL-17 signaling.

AS is known to increase Tγδ17 cells [42] and since these cells are one of the main producers of IL-17, they thereby propel inflammation and likely pathological new bone formation in the affected spinal entheseal compartment of AS [43]. Correspondingly, it is also shown that Piezo1-mediated mechanotransduction does have a role in entheseal pathological new bone formation as well by being abnormally upregulated in these affected entheseal compartments and ligaments of AS [44]. In summary, it seems that chronic spinal facet joint Piezo2 channelopathy and the resultant impaired Piezo2-Piezo1 crosstalk will eventually lead to IL-17-induced entheseal Piezo1 upregulation in a feed-forward, low-grade inflammatory manner in AS. As a result, Th17/Treg imbalance occurs, leading to uncontrolled Th17 production in AS [45] as could be witnessed in other autoimmune diseases, and seems to have a pivotal role in the cascade of inflammatory events leading to distressed ocular surfaces and dry [46,47] eyes. Furthermore, the upregulated Piezo1 might have a role in the higher differentiation of bone marrow stem cells towards osteoblasts. Noteworthy is that entheses are in close proximity to the bone marrow, and small holes are present at the enthesis-bone interface [48]. Indeed, Piezo1 is not only essential for bone formation, since knockout of Piezo1 in osteoblasts leads to disruption in osteogenesis [49], but also a regulator of stem cells’ fate determination [50]. Moreover, Piezo1 indeed has a role in the entheseal pathological new bone formation in AS, as mentioned before [44].

The downstream impact of this critical neuroimmune link has been demonstrated earlier, since the proposed impaired crosstalk between Piezo2 and Piezo1 is theorized to downregulate Piezo1 on corneal Langerhans cells (LC), leading to their activation in DED secondary to autoimmune diseases [36], like is observed in rheumatoid arthritis (RA) [51], systemic lupus erythematosus (SLE) [52] and AS [53], in addition to the aforementioned corneal nerve regeneration disruption in RA [36] and SLE [35]. Considering the aforementioned background, the current authors hypothesize that corneal nerve fibers go through similar pathological pathways leading to morphological and functional abnormalities, as was observed in other autoimmune conditions like RA [36] and SLE [35].

That being so, the purpose of the current study was to investigate the morphological signatures of lost functional remodeling and regeneration capacity of corneal sensory afferent fibers by in vivo confocal microscopy in AS. We followed an interdisciplinary approach along with our hypothesis, including, e.g., neuroscience, immunology, and rheumatology, to find clues to the long-unresolved autoimmune mechanism of AS. This approach might be permissive due to the evolutionarily conserved feature of Piezo channels, not to mention their role in axon regeneration [30] and also in the inhibition of axon regeneration [54].

## 2. Results

The demographics and clinical data of patients are summarized in Table 1. There was no significant difference in age between the control and patient groups. Among dry-eye parameters, tear production was lower and OSDI values were higher in the AS group.

### 2.1. Comparison of AS and Control Groups

The values for all confocal parameters for each group and the results of the statistical comparisons are summarized in Table 2. The most relevant findings are discussed below.

**Figure 1:**
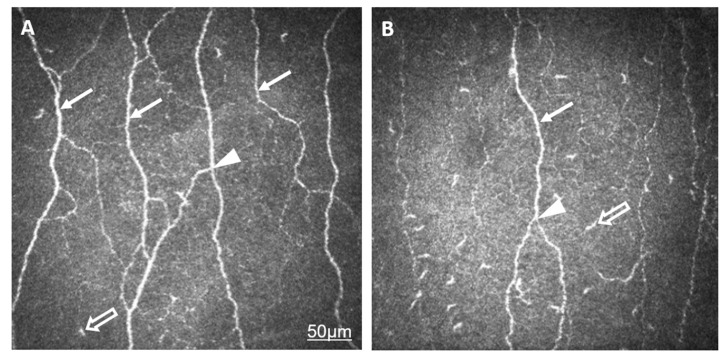
Representative in vivo confocal microscopic images of the corneal subepithelial nerves in a healthy eye (**A**) and the eye of a patient with AS (**B**). The lower density (CNFD) and reduced thickness and length (CNFL) of subepithelial nerve plexi (arrow) in AS compared to normal eyes are demonstrated. The number of branches (CNBD, CTBD, arrowhead) has decreased in AS. An increased number of Langerhans cells (empty arrow) is shown in the level of nerve plexi. The bar represents 50 µm.

Amongst the corneal subbasal nerve parameters Corneal nerve-fiber density (CNFD, the number of fibers/mm^2^), corneal nerve-branch density (CNBD, the number of branch points on the main fibers/mm^2^), corneal nerve-fiber length (CNFL, the total length of the nerves in mm/mm^2^), corneal nerve-fiber total branch density (CTBD, the total number of branch points/mm^2^), and corneal nerve-fiber area (CNFA, the total nerve-fiber area in mm^2^ per mm^2^) were lower in the AS group. All LC-related description values were higher in the AS group except peripheral LCM (Table 2, Figure 1).

### 2.2. Subgroup Analysis by Disease Activity

Among the AS group, there were 12 patients with positivity for human leukocyte antigen B27 (HLA-B27). We further examined the patient group by comparing the functional assays as well as the confocal variables in each subgroup concerned. Interestingly, we could not find a significant difference between these two subgroups in any of the variables examined (Table 3).

Similarly to our latest published work, we further divided the AS group according to the BASDAI score of the individual patients (BASDAI ≥ 4 vs. BASDAI < 4). Further analyses in BASDAI-related AS subgroups showed higher values in peripheral LC density in patients with higher BASDAI scores, while the CNFD values were significantly lower in this subgroup (Table 4).

### 2.3. Correlation Analysis

Correlation analysis revealed several correlations among general variables, dry eye parameters, corneal nerve descriptives, and LC distributions (Table 5). A positive correlation was found between disease duration and BASDAI score, while the Schirmer-test value had a negative correlation with the disease duration. A higher LIPCOF was observed in higher BASDAI cases. A negative correlation between TBUT and CTBD was detected. Schirmer was negatively correlated with peripheral LC Morphology, CNFL, We, and CRP, while positively correlated with CNFD. Surprisingly, none of the variables correlated with OSDI values. The density and morphology of central LCs were not dependent on any of the parameters. Peripheral LC density showed a negative correlation with the nerve fiber descriptives CNFD, CNBD, and CNFL, pointing to nerve damage depending on the presence of LC density. Peripheral LC morphology was negatively correlated with Schirmer, CNFD, CNBD, and CNFL.

CNFD was correlated positively with the Schirmer value and negatively with peripheral LCD and LCM. CNBD had a negative correlation with the peripheral LCD and LCM. Similar to CNFD, CNFL had a significant positive correlation with Schirmer, and CNFL was positively dependent on peripheral LCD and LCM. CTBD had a negative correlation with TBUT. CNFA was not correlated with any of the parameters.

## 3. Discussion

Ocular surface homeostatic mechanisms are dependent on precise intercommunicating arrangements of intact neuronal networks. The cornea and conjunctiva are densely innervated by the peripheral neurons of the trigeminal ganglion. These neurons form a subbasal nerve plexus within the cornea, whose ascending branches ramify extensively to terminate within the surface epithelial layers. The unimpaired neuroepithelial contact and their constant interactions provide for the maintenance of ocular surface homeostasis via mechanisms that are yet to be fully elucidated.

There is a growing body of evidence that resident immune cells of the ocular surface play a key role in orchestrating these delicate interactions through constant communication amongst them that may result in amplification or inhibition of the exaggerated immune reactions operating on the ocular surface in a diseased state. Indeed, vasoactive intestinal peptide (VIP) secreted by corneal nerves can down-regulate pro-inflammatory cytokines while up-regulating anti-inflammatory cytokines such as TGF-b [55,56]. The corneal epithelium constantly expresses vascular endothelial growth factor receptor-3 (VEGFR-3), which binds and thus inhibits angiogenic VEGF-C and VEGF-D, contributing to the normal immunohomeostatic environment. Resident corneal dendritic cells can respond to proinflammatory cytokines like TNF-α and IL-1, potentially initiated by NKT cells responding to stressed ocular surface epithelium and followed by other endogenous factors that cause antigen-presenting cells (APC) to traffic to the regional lymph nodes after maturation. It is important to note here that the aforementioned depletion of syndecan-1 might lead to lost regulation of NKT cells and the resultant chronic NKT cell depletion in AS. Since syndecan is needed for aggrecanase activation, it could lead to the accumulation of aggrecan, explaining the aggrecan-induced AS model in mice and the alleviation of mechanical load by tail suspension. Syndecans also affect axon regeneration and neurite outgrowth in different species, indicating the conserved role of these proteoglycans [57,58]. Syndecan-1 was also shown to stimulate adult neurogenesis [59]. Interestingly, higher serum syndecan-1 levels were observed in AS patients compared to healthy controls [60]. The extracellular domain of syndecan-1 can be cleaved by membrane-type matrix metalloproteinase-1 (MT1-MMP), leading to the so-called shedding that promotes cell migration [61], but shedding released into the serum could explain the elevated serum level as well. Since Piezo1 activation leads to the activation of MT1-MMP [62], this increased MT1-MMP activity might lead to increased shedding and high syndecan-1 levels in the serum.

Regulatory T cells (Tregs) can suppress naive T cell priming in draining lymph nodes (LN) through the secretion of TGF-b, which can affect cell-contact-dependent Treg/APC interactions [63]. Further to it programmed death-ligand (PD-L)1 and Fas ligand (FasL) ligation on activated T cells leads to the death of T cells [64]. In dry eye, mature APCs can initiate the expansion of IFN-g-secreting CD4þ T (Th1) and IL-17-secreting CD4þ T (Th17) cells in draining LNs. Furthermore, harmful effector T cells, especially Th1 and Th17 cells, can unrestrainedly migrate to the ocular surface because of dysfunctional Treg cells under the influence of increased levels of chemokines on the ocular surface, including CCL3/4/5, CXCR9/10 (for Th1 influx), and CCL20 (for Th17 influx) [56]. The increased levels of IFN-g and IL-17 from activated T cells on the ocular surface will eventually cause further corneal barrier disruption establishing a vicious circle of events where proinflammatory cytokine-dominated events prevail [65]. However, the current authors suggest that the initial corneal barrier disruption and immune cell migration are due to the Piezo2 channelopathy-induced impaired Piezo2-Piezo1 cross-talk.

Being a transparent tissue, the cornea provides us with a unique opportunity to gain insight into this delicate world of immuno-neuroepithelial crosstalk. In vivo corneal confocal microscopy (IVCM) is a non-invasive ophthalmic imaging technique that enables us to have insight into the cellular “world” of the cornea. In general, confocal microscopes are extensively used in various clinical settings and in experimental studies. Amongst the three IVCM devices that are commercially available, there is only one brand, the Heidelberg Retina Tomograph II or III with the Rostock Corneal Module (RCM) (Heidelberg Engineering, GmBH, Germany), that designs the Laser Scanning Confocal Microscope (LSCM) we employed due to the high-contrast images it provides [66]. Over the past few years, several studies have investigated and proven the usefulness of IVCM in the detection of minute changes in the cornea in various systemic disorders. One particular interest that drew attention lately was related to the corneal neuronal network changes in systemic ailments like diabetes and neurogenerative diseases [67,68]. Indeed, IVCM is currently considered a reliable, reproducible, and quantitative diagnostic method for early detection of diabetic neuropathy through in vivo observation of the corneal nerves. Also, there are studies that showed nerve fiber alterations and an increase in dendritic cell number in patients with active COVID-19 [69].

Undeniably, IVCM with an axonal resolution of four microns is far more than capable of capturing intimate cell-cell or cell-neuron interactions that are of paramount importance to shaping our understanding of the neuro-immunological milieu. Reports are scarce using IVCM to show the correlation between the immune and nervous systems in the human cornea for certain diseases, like AS. In the current study, OSDI was used to evaluate the symptoms of ocular irritation and the effects it has on the patient’s vision. It is important to note that earlier findings showed that corneal nerve length was negatively correlated with sensitivity to light and nerve width was positively correlated with the OSDI score, painful eyes, and blurred vision [70]. The negative correlation between TBUT and CTBD, tear production, and CNFL could be interpreted as a direct consequence of loss of tear film-related osmotic pressure over the cornea; however, Ferdousi et al. failed to find a correlation between dry eye syndrome and TBUT or corneal nerve morphology [71].

Morphology and function do not always correlate, but even subclinical corneal nerve loss can be clinically significant, as for every 1 mm/mm^2^ loss in CNFL, there is a 0.61 °C reduction in the cold detection threshold and 1.78% lower heart rate variability in type 1 diabetes [72]. Not only the CNFL but all the corneal nerve parameters were lower than normal in AS patients, which is in line with other studies investigating corneal nerve alterations in systemic conditions like diabetes and systemic inflammatory conditions [73]. It is noteworthy that altered metabolism, like diabetes, and altered inflammatory signaling could contribute to distal axonal degeneration [74]. Suitably, terminal arbor degeneration is also theorized to evolve due to Piezo2 channelopathy [17], as Piezo2 channelopathy could enhance the deregulation of the autonomic nervous system [17].

Peripheral LC density showed a negative correlation with the nerve fiber descriptives of CNFD, CNBD, and CNFL, which highlights the ever-changing interactions between immune cells and neural components in the cornea. We know from previous studies that inflammation alters corneal sensory nerve activity where polymodal nociceptors are sensitized [75], generating spontaneous pain and hyperalgesia experienced during inflammation like in DED (69).

Noteworthy is that amongst the corneal sensory neurons, the polymodal nociceptors are the most numerous (Aδ and C fibers), comprising mechano-, thermal-, and chemical nociceptors. These fibers are different from the pure mechano-nociceptors that are activated only by soft mechanic forces in the order of magnitude close to that required to damage corneal epithelial cells and make up approximately 15% of corneal nerve fibers [53,54]. These mechano-nociceptors express Piezo2 channels [76] which, along with Piezo1, are the main channels playing crucial roles in maintaining a stress-free ocular surface. We know from previous studies that DED can be brought on by repetitive microinjury to the ocular surface in the form of tear hyperosmolarity, resulting in inadequate responses from the decalibrated and probable injured mechanonociceptors. Recently, it was hypothesized that autologous Piezo2 channelopathy and its reinjury could be the initiating steps toward DED development [10]. Evidence is emerging that DED is not only associated with a higher density of immune cells in the cornea, but there is a significant reduction of corneal nerve-related parameters that are likely to contribute to DED [77] evolution.

The current authors interpreted and showed earlier that the autologous Piezo2 channelopathy-induced impaired Piezo2-Piezo1 crosstalk could be the reason why neural regeneration of the cornea is disrupted in DED secondary to RA and SLE [35,36]. In line with earlier findings, the current study also exhibits on IVCM that neural regeneration is disrupted in DED secondary to AS.

One of the most prevailing theories of AS pathogenesis is that HLA-B27 heavy chain misfolding gradually induces endoplasmic reticulum stress, leading to altered antigen presentation and abnormal natural killer cell and autoreactive T cell activation [78]. A flare-up of this mechanism could prevail upon dysbiosis or mechanical stress, resulting in a low-grade pro-inflammatory microenvironment. Besides this, the aforementioned Piezo2 channelopathy theory proposes that the microinjury of Piezo2 ion channels in proprioceptive terminals is one principal gateway from physiology to pathophysiology [10], not to mention that it could open a potentially chronic inflammatory pathway upstream depending on reinjury and genetic and environmental risks [10]. According to this Piezo2 channelopathy theory, Piezo2 ion channels should be considered as homeostatic gatekeepers of peripheral sensory neuron terminals in a compartmental micro-milieu, whereas Piezo1 ion channels are the homeostatic gatekeepers of the surrounding peripheral cells. This Piezo2 channelopathy could be one ignitor of the so-called neural circuit-based inflammatory reflex [79], with a first line disbalanced activated NKT cell response [37]. The current authors propose that the mechano-energetic impairment of these Piezo2-containing sensory terminal gateways is one pathway to invoke the so-called gateway reflexes on the chronic path. The gateway reflexes are neural circuit-based protective systems to maintain neuroimmune homeostasis [80]. The chronic inducement of these inflammatory reflexes could mobilize autoreactive T cells in the central nervous system (CNS), regardless of selective endothelial barrier protection [80]. Invoking this mechanism requires noradrenalin, which is associated with an Interleukin-6 (IL-6) amplifier, leading to non-immune endothelial nuclear factor-KB (NF-κB) production due to environmental or experimental stimuli [81]. However, the initiation of this reflex activation is suggested to require a point of primary damage, and that is the proposed, but initially pain-free, Piezo2 channelopathy [10,12]. Furthermore, the chronification of this sensory terminal Piezo2 microinjury is suggested to be the chronic form of Piezo2 channelopathy [10,17] and that is the equivalent of the aforementioned gateway reflex activation, according to the authors of this manuscript. It is notable that the proposed transient proprioceptive terminal Piezo2 channelopathy is associated with NF-κB activation [20,37] and could evolve during allostasis [82]. These acute stress-related microinjuries on learning and memory-associated proprioceptive neural pathways could lead to the specific sympathetic activation of the IL-6 amplifier on the chronic path. Noteworthy is that Piezo2 channelopathy is also suggested to be linked to the TLR4/IL-6/TNF-α pathway [37]. Moreover, Piezo2 channelopathy is theorized to be the microinjury that increases the IL-6 level [13,20], so it could be considered to be the IL-6 amplifier. In addition, it is important to note that the allostatic acute stress is theorized to be invoked by osteocalcin [2] or oxytocin [17], and indeed recent research shows that Piezo1 modulation has a role in the upregulation of oxytocin synthesis [83]. In summary, the overstimulation of microenvironmental (e.g., muscle spindle, Merkel cell-neurite complex, encapsulated mechanoceptor neuron terminals in the facet joints) Piezo1-Piezo2 mechanostransduction under allostasis could lead to Piezo2 channelopathy at proprioceptive terminals or in sensory terminals contributing to proprioception, hence resulting in the impairment of the Piezo2-Piezo1 crosstalk in these compartmental micromilieus and the initiation of inflammatory reflexes.

Correspondingly, we hypothesize that the initiating bi-phasic and bi-compartmental (facet joints and entheseal compartments) Piezo2 channelopathy, in an analogous way like in DOMS [20], induced impaired Piezo2-Piezo1 crosstalk will lead to transcription activation, and if underlying mutations, like allelic variants of the HLA-B27 genotype, are present, then AS could evolve with Piezo2 channelopathy chronification, and that is the suggested activated gateway reflex, or the tertiary injury phase of Piezo2 channelopathy [10]. Noteworthy is that the soleus muscles have a dominant role in anti-gravitational and postural control, not to mention they largely contribute to the gravity gateway reflex [80]. Even more important than the pseudounipolar proprioceptive sensory neurons of the soleus arrive at the L5 spinal segment [80] and that is the vicinity where mechanical stress and lost mechanotransductory signaling are implicated in AS [21]. It is known that, under experimental autoimmune pathological conditions, autoreactive T cells could invade specific blood vessels at the L5 level [21]. Moreover, it is theorized that proprioceptive terminal Piezo2 channelopathy could induce a secondary protective preprogram to increase postural control, shock attenuation, and anti-gravitational protection due to a switch, later called “mis-wire” by Espino et al., [84]. However, this protection is costly segmentally in terms of neuro-energetics, therefore it could suck neuro-energetic resources from other proprioceptive feedback pathways [3,12,17,19,20]. The authors of this manuscript describe the stiffness experienced in AS due to this Piezo2 channelopathy-induced switch/miswiring of proprioception. This stiffness is alleviated during the day, probably due to the unloading of the miswired proprioceptive feedback by concentric contractions [3,85]. Indeed, it has been long observed that morning stiffness improves throughout the day.

Another gateway reflex, namely the light gateway reflex, is observed to be activated in HLA-B27 spondyloarthropathies with intraocular inflammation [80]. The uveal tract supplies the intraocular part of the eye, and the activation of the light gateway reflex could induce autoreactive T cell invasion and idiopathic intraocular inflammation secondary to systemic autoimmune diseases [80], like in AS. Current authors suggest that the impaired Piezo2-Piezo1 crosstalk also has a role in the light gateway reflex activation in AS, leading to uveitis; however, this mechanism is not the subject of this manuscript. Nevertheless, it has been theorized that chronic Piezo2 channelopathy in autoimmune diseases, like RA and SLE, could be linked with autologous corneal Piezo2 channelopathy leading to increased DED susceptibility secondary to these autoimmune conditions, and the same is suspected in our current AS findings.

The genetic risk of AS is long known, especially the aforementioned HLA-B27 link to AS [86]. The proposed Piezo2 channelopathy-associated transcription activation brings a new interpretation to the underlying molecular mechanism since chronic microinjury of these ion channels is suggested to lead to upregulated unfolding protein reaction (UPR) and protein misfolding [12] in support of the aforementioned prevailing AS mechanism theory. Noteworthy that the activation of UPR is within homeostasis in association with the transient form of the proposed Piezo2 microdamage [12]. However, an underlying genetic predisposition could derail this process since Piezo2 channelopathy is also suggested to be a principal transcription activator; therefore, it could reveal pathogenic gene variants during this microdamage [12]. Accordingly, the proposed chronic Piezo2 channelopathy-induced upregulation of endoplasmic reticulum UPR could increase protein misfolding due to changes in its peptide binding characteristics of the allelic variants of the HLA-B27 genotype, as was suggested by Voruganti et al. earlier [87]. Noteworthy is that research is emerging that Piezo is indeed responsible for buffering sarcoplasmic reticulum, or endoplasmic reticulum, stress through intracellular calcium handling [88]. Moreover, the Piezo2 channelopathy theory proposes that during allostatic stress, if the Piezo2 channels are microinjured, they cannot sustain their inactivated gating mechanism, hence they become leaky to unbalanced subthreshold calcium currents [10,17] and intracellular stress buffering is impaired, leading to the aforementioned upregulation of UPR.

Other interesting gene variants in AS about Piezo2 channelopathy are the prostaglandin EP4 receptor (EP4)-coding PTGER4 gene and the endoplasmic reticulum aminopeptidase-coding ERAP1 gene [89]. Involved AS spines have abundantly expressed EP4 receptors in entheses [89]. Noteworthy, that EP4 is one receptor for prostaglandin E2 (PGE2), and PGE2 is involved in the excitatory pathway of mechanotransduction by being a neuromodulator of encapsulated large fiber bone afferents, contributing to proprioception [19]. The current authors suggest that the upregulated expression of EP4 could lead to a higher risk of prolonged mechanotransductory Piezo2 microdamage of encapsulated large fiber bone afferents in the facet joint that are contributing to proprioception, hence leading to higher susceptibility to AS development in the presence of the pathogenic PTGER4 gene variant. Moreover, ERAP1 polymorphism could derail the activated UPR towards misfolding HLA-B27 antigens in HLA-B27-positive AS individuals. Even more importantly, in reference to DED secondary to AS, GPR65 could be one critical gene in autoimmune pathogenesis since its presence increases IL-17 in the subpopulation of certain Th17 cells [87].

A pain spectrum, even including neuropathic pain, is often associated with AS over time [90], like in the case of DED [10]. A recent systematic review is exposing the issue of whether central sensitization of pain could be sustained as an autonomous pain generator in the absence of peripheral input [91]. The investigation concluded that peripheral input could be crucial and needed as a driver [91]. The phenomenon long known in humans is that mechanical hyperalgesia and allodynia invoked by tissue injury could be experienced in neighboring uninjured tissues [92], like in the cases of DOMS [17,20] and DED [10]. The TRPV1 ion channel is an essential signaler of this phenomenon [20,92] However, it is postulated that this is not true in the absence of Piezo2 primary damage [17,20,92]. Indeed, loss-of-function mutations in Piezo2 cause loss of pain and sensitization, as mentioned earlier [27], and Piezo2-containing nociceptors are involved in mechanical sensitization [93]. Moreover, activated TRPV1 channels have a role in the inhibition of Piezo channels [94]. It is important to note that wide dynamic range (WDR) neurons may have a central role in the central sensitization of this mechanism in the spinal cord [12,95]. WDRs, with their distinctive feature of reflecting upon the gate control theory of pain [96], not only provide an explanation for how painful DOMS and painless ALS could be initiated by the same proprioceptive terminal Piezo2 channelopathy [12], but might also explain the flare-ups and continuum to neuropathic pain of AS and other autoimmune diseases. Not to mention that, after all, there might be a link between AS and ALS even in the absence of anti-TNF-α treatment [97].

Finally, the current study has some limitations. On one hand, mainly male subjects were included in the AS group due to the natural course of the disease. The gender difference in the control group could lead to interpretation complications. In order to solve the difficulty, we did not analyze the gender-related differences. On the other hand, AS is a variable disease with the involvement of different organs at different stages. Hence, the therapy might influence the measured microstructural and dry eye characteristics of the eyes. Accordingly, the correlations between the severity of ocular symptoms and disease activity were carefully analyzed in the current study.

## 4. Materials and Methods

Our study was a cross-sectional, comparative investigation of 24 patients with AS and 35 healthy volunteers who served as control subjects. This study received approval from the Central Ethics Committee of Hungary (ETT TUKEB, 15410-2/2011-EKU), and all participants provided written informed consent for the examinations. The entire research process adhered to the principles of the Declaration of Helsinki.

Patients with AS were diagnosed in accordance with the revised New York criteria [98]. All AS patients were under consistent treatment with anti-TNF-α therapy, ensuring that the duration of the same anti-TNF-α medication was more than 3 months. Among the AS patients, fifteen (62%) additionally received nonsteroidal anti-inflammatory drugs, while six (25%) were prescribed sulphasalazine. The Bath Ankylosing Spondylitis Disease Activity Index (BASDAI) was computed during the ocular examination. BASDAI, a six-item questionnaire assessing disease activity, serves as a prominent and extensively employed tool for evaluating the activity of AS [99]. Participants who exhibited any obvious ocular symptoms necessitating specific ophthalmic care, individuals with diabetes mellitus, a history of previous eye surgery, uveitis within the year preceding the examination, glaucoma, congenital, mechanical, or toxic corneal injury, or any illnesses leading to corneal edema, haze, or scars were excluded from this study. Since Sjögren-related hypolacrimation can lead to severe dry eye and, thus, significant changes in the morphology of the subbasal nerve plexus (SBNP) [100,101], patients diagnosed with secondary Sjögren’s syndrome based on the revised European criteria for Sjögren’s syndrome were also excluded. Secondary Sjögren’s syndrome could evolve with extreme dryness of the eye and mouth as a result of established connective tissue diseases. Correspondingly, the exclusion criteria were as follows for secondary Sjögren’s syndrome: positivity of item I (ocular symptoms) or item II (oral symptoms), in addition to any combination of two items from III (ocular sign), IV (histopathology), and V (salivary gland involvement) according to the “Revised international classification criteria for Sjögren’s syndrome” table [100]. Nevertheless, patients with Schirmer test results of less than 5 mm/5 min were not excluded automatically from this study as long as their results from other functional tests and histology were normal, and no anti-Ro (SSA) or anti-La (SSB) antibodies were detected.

Initially, the signs and symptoms related to dry eye were assessed. To evaluate the subjective ocular discomfort associated with dry eye symptoms, the OSDI questionnaire was utilized [102]. Then, all patients underwent a conventional slit lamp biomicroscopic examination. The grade of the lid-parallel conjunctival folds (LIPCOF) was evaluated according to a previously reported method [103]. The Tear Break-Up Time (TBUT) was assessed by applying fluorescein vital dye to the ocular surface, and the time between the last blink and the first appearance of corneal black spots was measured. The average value from three consecutive measurements was considered the TBUT. Tear production was evaluated using the Schirmer strip (Haag-Streit UK Ltd, Bishop’s Stortford, UK). The strip was gently placed into the temporal part of the lower cul-de-sac without anesthesia, and readings were taken after 5 min.

Next, IVCM was conducted. All patients underwent examination using the Rostock Cornea Module of the Heidelberg Retina Tomograph-III (Heidelberg Engineering GmbH, Heidelberg, Germany), equipped with the built-in software Heidelberg Eye Explorer version 1.5.10.0. Prior to the examination, topical anesthesia was performed with one drop of oxybuprocaine-hydrochloride (Oxybuprocaine-Humacain 0.4%, Human Pharmaceuticals, Gödöllő, Hungary) instilled on the ocular surface. A sterile disposable polymethyl methacrylate cap (TomoCap^®^, Heidelberg Engineering GmbH. Heidelberg, Germany) was used to cover the microscope lens. To maintain airless contact, artificial tear gel (0.2% carbomer, Vidisic^®^, Chem.-pharm. Fabrik GmbH, Brunsbütteler, Berlin, Germany, Bausch&Lomb) was applied as a coupling medium. En-face, images were captured at the level of SBNP (at 40–60 mm depth from the ocular surface) using a 400 µm field of view lens. All examinations were performed under consistent light conditions.

Regarding the evaluation of the IVCM images, the quantitative analysis of the density and morphology of LCs both in the center and in the corneal periphery at 6 o’clock [104], along with the characterization of the central SBNP morphology, was performed. For LCs analysis, five representative images were selected, and the Langerhans cell density (LCD) was calculated with the in-built semi-automatic CellCount function of the device (cell number/mm^2^). Langerhans cell morphology (LCM) was assessed using a grading scale ranging from 0 to 3. A score of 1 indicated LCs without dendrites, a score of 2 denoted LCs with small processes, and a score of 3 represented LCs with extensive processes. A score of zero (0) indicated the absence of LCs in the area of interest. Each LC was individually scored, and the mean score was calculated [53].

The ACCMetrics V.2 software was utilized to assess the morphometric parameters of the SBNP [45]. A series of images (between 5 and 10) representing the SBNP were collected, and the software automatically quantified the following parameters using multiple analysis modes: corneal nerve fiber density (CNFD, the number of fibers/mm^2^), corneal nerve fiber length (CNFL, the total length of nerves mm/mm^2^), corneal nerve fiber total branch density (CTBD, the total number of branch points/mm^2^), and corneal nerve fiber width (CNFW, the average nerve fiber width mm/mm^2^). Statistical procedures: variables for the selected samples were presented as means and standard deviations. For the purpose of selecting an adequate statistical procedure and considering the distribution of the measured data, Shapiro–Wilk’s W test of normality was calculated. For the comparison of the measured datasets, an independent sample t-test and a Mann–Whitney U test were performed based on normality. For the determination of the correlation between the datasets, Spearman correlation was performed. Statistical calculations were executed with Statistica version 12 software (TIBCO Software Inc., Palo Alto, CA, USA) and JASP software (jasp-stats.org, installed version 0.16). The significance level was determined in all cases at *p* < 0.05.

## 5. Conclusions

The results of our confocal microscopy study indeed demonstrated that corneal sensory afferents show reduced regeneration activity in DED secondary to AS. In light of this finding, the current study adds to emerging research in support of the neurocentric instinct of Bekhterev in reference to AS, not to mention his other instinct of investigating reflexes. After all, stretch reflexes, with the Piezo2 contribution, play an important role in postural control and erection in humans, not to mention eye movements and vision. The microinjury of these principal proprioceptive channels could initiate inflammatory reflexes acutely and gateway reflexes on the chronic path, like the gravity gateway reflex in AS. One telling symptom will be impaired proprioception with underlying proprioceptive miswiring, where the cause is theorized to be Piezo2 channelopathy.

The findings of this study may facilitate a better understanding of the mechanisms leading to neural microdamage and inflammation in the cornea of dry eye and AS. Correspondingly, the potential pharmacological improvement of corneal nerve regeneration might result in decreased dry eye symptoms for the patients in the long run. The technique applied in the current study could be an applicable tool to measure and follow up on the efficacy of the treatments.

## Figures and Tables

**Table 1:** Demographic and clinical data in the control and AS groups. Data are shown in mean ± SD values: AS disease activity index (BASDAI); tear film break-up time (TBUT); lid parallel conjunctival folds (LIPCOF); and ocular-surface disease index (OSDI). * *p* < 0.05 Independent Samples T-test/Mann–Whitney test.

	Control	AS	*p*
No. of patients	35	24	NA
No. of eyes	35	24	NA
Age (years)	44.2 ± 19.94	41.9 ± 9.8	0.907
Gender (male/female)	11/18	1/28	NA
AS duration (years)	-	9.9 ± 4.5	NA
BASDAI	-	3.2 ± 2.11	NA
LIPCOF	1.02 ± 0.7	0.95 ± 0.62	0.0
TBUT (s)	11.28 ± 3.0	11.47 ± 4.63	0.85
Schirmer test (mm/5 min)	12.1 ± 2.9	8.4 ± 8.11	0.016 *
OSDI	8.8 ± 6.56	21.52 ± 15.44	≥0.001 *

**Table 2:** Comparison of AS and Control groups. Data are shown in mean ± SD values. * *p* < 0.05 Independent Samples T-test/Mann–Whitney test.

	Control	AS	*p*
CNFD	19.32 ± 7.87	13.59 ± 6.58	0.005 *
CNBD	25.77 ± 17.2	13.38 ± 7.82	0.002 *
CNFL	13.4 ± 3.82	10.43 ± 3.77	0.005 *
CTBD	44.2 ± 24.1	28.06 ± 12.4	0.005 *
CNFA	0.006 ± 0.002	0.005 ± 0.001	0.03 *
Central LCD	23.56 ± 25.37	77.57 ± 41.94	≥0.001 *
Peripheral LCD	75.52 ± 33.03	128.25 ± 56.33	≥0.001 *
Central LCM	1.0 ± 0.68	1.71 ± 0.69	≥0.001 *
Peripheral LCM	2.34 ± 0.54	2.53 ± 0.59	0.185

**Table 3:** Subgroup analysis HLA-B27. Mean and SD values for the confocal microscopic findings of the AS subgroups. For abbreviations, see the text. * *p* < 0.05 Independent T-test/Mann–Whitney test.

	HLA-B27 +	HLA-B27 −	*p*
No. of patients	12	12	NA
No. of eyes	12	12	NA
Age (years)	42.7 ± 7.6	43.1 ± 8.7	
AS duration (years)	8.4 ± 3.3	11.4 ± 5.1	0.104
BASDAI	3.0 ± 1.2	3.4 ± 2.7	0.670
ESR (We)	23.4 ± 17.9	23.5 ± 20.7	0.983
CRP	15.9 ± 18.7	18.6 ± 16.6	0.714
LIPCOF	0.92 ± 0.52	1.00 ± 0.74	0.752
TBUT (s)	11.5 ± 5.0	11.4 ± 4.5	0.949
Schirmer test (mm/5 min)	9.2 ± 8.2	7.6 ± 8.4	0.644
OSDI	23.9 ± 11.7	19.5 ± 18.2	0.509
CNFD	14.4 ± 5.4	12.8 ± 5.4	0.960
CNBD	13.3 ± 7.3	13.5 ± 8.7	0.857
CNFL	10.6 ± 3.2	10.3 ± 4.4	0.879
CTBD	2.76 ± 11.7	28.5 ± 13.7	0.312
CNFA	0.005 ± 0.002	0.006 ± 0.001	0.618
Central LCD	79.3 ± 36.7	75.8 ± 48.2	0.843
Peripheral LCD	141.4 ± 53.5	115.1 ± 58.2	0.261
Central LCM	1.8 ± 0.7	1.6 ± 0.7	0.387
Peripheral LCM	2.7 ± 0.5	2.4 ± 0.7	0.308
Endothelium	2645 ± 566	2627 ± 472	0.935

**Table 4:** Subgroup analysis BASDAI. Mean and SD values for the confocal microscopic findings of the AS subgroups. For abbreviations, see the text. * *p* < 0.05 Independent T-test/Mann–Whitney test.

	BASDAI ≤ 4	BASDAI > 4	*p*
No. of patients	17	7	NA
No. of eyes	17	7	NA
Age (years)	41.5 ± 9.4	45.4 ± 10.3	0.408
AS duration (years)	8.7 ± 3.9	13.5 ± 4.7	0.026 *
BASDAI	2.2 ± 1.2	5.9 ± 1.6	<0.001 *
ESR (We)	18.0 ± 13.7	32.2 ± 22.7	0.082
CRP	12.3 ± 13.5	23.5 ± 15.2	0.105
LIPCOF	0.82 ± 0.64	1.33 ± 0.52	0.093
TBUT (s)	12.5 ± 4.5	9.0 ± 4.1	0.133
Schirmer test (mm/5 min)	10.2 ± 8.5	4.3 ± 5.8	0.137
OSDI	21.2 ± 14.6	17.6 ± 17.2	0.644
CNFD	15.2 ± 6.2	8.1 ± 6.1	0.025 *
CNBD	14.5 ± 7.5	10.5 ± 8.6	0.305
CNFL	10.8 ± 3.6	8.6 ± 3.7	0.213
CTBD	27.5 ± 12.3	29.4 ± 13.6	0.756
CNFA	0.005 ± 0.001	0.005 ± 0.002	0.755
Central LCD	73.8 ± 45.1	97.0 ± 23.2	0.246
Peripheral LCD	109.1 ± 44.2	175.6 ± 63.6	0.010 *
Central LCM	1.8 ± 0.6	1.5 ± 0.8	0.334
Peripheral LCM	2.4 ± 0.6	3.0 ± 0.0	0.321
Endothelium	2559 ± 510	2720 ± 451	0.503

**Table 5:** Summary of significant correlations among parameters: + represents a significant positive correlation; − represents a significant negative correlation between variables. *p* < 0.05 (Spearman correlation).

	AS Duration	BASDAI	ESR (We)	CRP	LIPCOF	TBUT	Schirmer Test	CNFD	CNBD	CNFL	CTBD	CNFA	Peripheral LCD	Peripheral LCM
AS duration		+					−							
BASDAI	+				+			−						
ESR (We)				+			−							
CRP			+				−							
LIPCOF		+												
TBUT											−			
Schirmer test	−		−	−				+		+				−
CNFD		−					+						−	−
CNBD													−	−
CNFL							+						−	−
CTBD						−								
Peripheral LCD								−	−	−				
Peripheral LCM							−	−	−	−				

## Data Availability

The data presented in this study are available on request from the corresponding author.

## Abbreviations

ALS	amyotrophic lateral sclerosis
APC	antigen-presenting cell
AS	ankylosing spondylitis
axSpA	axial spondyloarthritis
BASDAI	AS disease activity index
CNS	central nervous system
CNBD	corneal nerve branch density
CNFA	corneal nerve fiber area
CNFD	corneal nerve fiber density
CNFL	corneal nerve fiber length
CTBD	corneal nerve fiber total branch density
CRP	C reactive protein
DED	dry eye disease
DOMS	delayed onset muscle soreness
EP4	prostaglandin EP4 receptor
HLA-B27	human leukocyte antigen B27
IL-6	Interleukin-6
IL-17	Interleukin-17
L5	lumbar 5
LC	Langerhans cells
IVCM	In vivo corneal microscopy
LCD	Langerhans cell density
LIPCOF	lid parallel conjunctival folds
LN	lymph node
MT1-MMP	membrane type matrix metalloproteinase-1
NKT cell	natural killer-T cell
NKT17	IL17 producing NKT
NF-κB	nuclear factor kappa B
OSDI	ocular surface disease index
PGE2	prostaglandin E2
PD-L	programmed death-ligand
RA	rheumatoid arthritis
SBNP	subbasal nerve plexus
SLE	systemic lupus erythematosus
TBUT	tear film break-up time
Treg	T regulatory cell
Tγδ17	γδ17 cell
UPR	unfolding protein reaction
VIP	vasoactive intestinal peptide
WDR	wide dynamic range
